# Mendelian Randomization Analysis Reveals Statins Potentially Increase Amyotrophic Lateral Sclerosis Risk Independent of Peripheral Cholesterol-Lowering Effects

**DOI:** 10.3390/biomedicines11051359

**Published:** 2023-05-04

**Authors:** Wenjing Wang, Linjing Zhang, Kailin Xia, Tao Huang, Dongsheng Fan

**Affiliations:** 1 Department of Neurology, Peking University Third Hospital, Beijing 100191, China; 2 Beijing Key Laboratory of Biomarker and Translational Research in Neurodegenerative Diseases, Beijing 100191, China; 3 Key Laboratory for Neuroscience, National Health Commission/Ministry of Education, Peking University, Beijing 100191, China; 4 Department of Epidemiology and Biostatistics, School of Public Health, Peking University, Beijing 100191, China; 5 Key Laboratory of Molecular Cardiovascular Sciences, Peking University, Ministry of Education, Beijing 100191, China

**Keywords:** amyotrophic lateral sclerosis, statins, mendelian randomization, causality, Hydroxymethylglutaryl-CoA reductase inhibitors, lipids, cholesterol, motor neurons, risk factors, medicine

## Abstract

Background: Observational studies suggest that statins may affect amyotrophic lateral sclerosis (ALS). However, they are limited by confounding and reverse causality biases. Therefore, we aimed to investigate the potential causal associations between statins and ALS using a mendelian randomization (MR) approach. Methods: Two-sample MR and drug-target MR were performed. Exposure sources included GWAS summary statistics of statin use, low-density-lipoprotein cholesterol (LDL-C), HMGCR-mediated LDL-C and LDL-C response to statins. Results: Genetic predisposition to statin medication was associated with increased ALS risk (OR = 1.085, 95% CI = 1.025–1.148, *p* = 0.005). After removing SNPs significantly associated with statin use from the instrumental variables (IVs), LDL-C-related higher ALS risk was absent (before removing: OR = 1.075, 95% CI = 1.013–1.141, *p* = 0.017; after removing: OR = 1.036, 95% CI = 0.949–1.131, *p* = 0.432). HMGCR-mediated LDL-C (OR = 1.033, 95% CI = 0.823–1.296, *p* = 0.779) and blood LDL-C response to statins (OR = 0.998, 95% CI = 0.991–1.005, *p* = 0.538) had no association with ALS. Conclusions: Here, we show that statins may be a risky exposure that increases ALS risk independent of the lowering effect of LDL-C in peripheral circulation. This provides insights into ALS development and prevention.

## 1. Introduction

Amyotrophic lateral sclerosis (ALS) is a fatal and progressive motor neuron disease without a cure. It is characterized by the degeneration of both upper and lower motor neurons, which leads to rapidly progressive paralysis culminating in death from respiratory failure [1,2]. Approximately 10% of ALS cases are familial (inherited), with the remaining 90% of cases being sporadic in origin, indicating that exposure plays an important role in the development of ALS. Compelling epidemiological studies have shown that environmental factors (e.g., neurotoxins, occupations, metals, pesticides, and viruses) and lifestyle factors (e.g., smoking, lower body mass index, vigorous physical activity, and unhealthy diets) are associated with an increased risk of ALS [3,4,5,6]. These exposure factors can trigger motor neuron degeneration via different biological mechanisms (e.g., neuroinflammation and mitochondrial impairment) [7,8,9]. However, beyond that, medication use for treatments is a kind of unique exposure that is artificial and iatrogenic. Identifying the causal pharmacological effects of medication exposure in ALS is important to provide insights into ALS development and make clinical decisions in ALS management [3,4].

Statins, also known as HMG-CoA reductase (HMGCR) inhibitors, commonly used to lower cholesterol [10], have raised concerns regarding their potential role in developing ALS. Statins inhibit many intracellular survival signaling pathways, which may harm to motor neurons. Statins inhibit HMG-CoA reductase, the rate-limiting enzyme that converts HMG-CoA to mevalonate, resulting in the synthesis of cholesterol and isoprenoids. Thus, statins not only decrease cholesterol synthesis, but also inhibit mevalonate-mediated cholesterol-independent effects. Both statins inhibited cholesterol, and mevalonate can activate intracellular survival signaling pathways to sustain cell survival. Compared with the cell biology of ALS, which involves death of motor neurons, cancer cell biology is characterized by uncontrolled cell proliferation. Interestingly, statins are widely reported to inhibit various types of cancer, including breast cancer, colorectal cancer, prostate cancer, etc. [11,12,13]. Indeed, some observational studies have already shown that statin use may be associated with an increased development risk of ALS [14,15,16,17]. However, observational studies are often underpowered and are subject to confounding and reverse causation, making causal inference quite difficult. Conducting randomized controlled trials (RCT) is challenging, as such a study seems to be unethical. In addition, they require long-term follow-ups and are expensive to carry out [18]. Therefore, it is desirable to determine the causal relationship between statin medication and ALS using potentially powerful statistical tools.

A powerful statistical tool to estimate the causal effects of a modifiable risk factor on a disease is Mendelian randomization (MR). This method relies on the principle of Mendel’s laws of inheritance, which employ single-nucleotide polymorphisms (SNPs) as unconfounded proxies (instrumental variables) for exposures, and then uses these genetic variants to estimate the causal effect of the exposure on outcomes of interest. MR analysis depends on three assumptions for the genetic variant used as an instrumental variable (IV). First, IV is associated with the exposure or risk factor of interest (the relevance assumption). Second, IV is independent of confounding factors (the independence assumption). Third, IV only affects the outcome or disease through the exposure or risk factor of interest (the exclusion restriction assumption) [19,20]. In this way, MR minimizes the biased results arising from confounding or reverse causation in observational studies, and it is similar to RCTs because the two alleles of an SNP are randomly segregated in the process of gamete formation [19]. MR is a cost-effective analytic tool for assessing causal effects when RCTs are unethical or impractical [21,22]. Therefore, the current issue addressed in this study is using an MR approach to assess the potential causal associations between statin use and ALS risk. Due to the blood–brain barrier (BBB), cholesterol cannot move between the central nervous system (CNS) and the rest of the body. As such, cholesterol metabolism in the CNS is proposed to operate autonomously. Lipophilic statins can cross the BBB, which means that we need to divide statin functions between two locations: the CNS or peripheral circulation [23]. In this study, we used available genome-wide association studies (GWAS) and adopted two-sample MR and drug-target MR approaches to systematically explore the statins’ total effects and low-density-lipoprotein cholesterol (LDL-C) lowering effect in the periphery on ALS, which may identify a medication exposure that triggers ALS, helping to make clinical decisions in ALS management.

## 2. Materials and Methods

**Figure 1:**
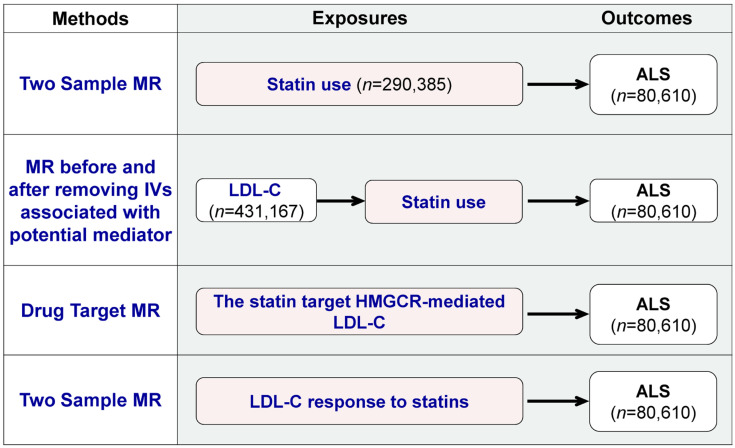
Overview of the study and Mendelian randomization (MR) model. The figure provides an overview of the four MR tests used to investigate the pharmacological effects of statins on ALS. The tests are: (1) two-sample MR to assess the association between statin use and ALS; (2) comparison of the association between LDL-C and ALS before and after removing SNPs associated with statin use; (3) drug-target MR to determine the statin target HMGCR-mediated LDL-C and ALS relationship; and (4) two-sample MR to analyze the relationship between the blood LDL-C response to statins and ALS.

As shown in Figure 1, two-sample MR and drug-target MR were performed to systematically explore the statins’ total effect and LDL-C lowering effect in the periphery on ALS. Two-sample MR is a commonly used MR method, in which the exposure and outcome data are obtained from two separate datasets, typically from summary results provided by the consortia of GWAS [20,24]. Drug-target MR is a variant of MR that can demonstrate the impact of modifying factors (such as circulation LDL-C) through specific therapeutic targets (such as the statin target: HMGCR) on long-term disease outcomes [25,26]. Four MR tests were designed. First, two-sample MR was used to assess the associations between statin use and ALS. Second, we compared the associations between LDL-C and ALS before and after removing SNPs associated with statin use. Third, drug-target MR was applied to reveal the statin target: HMGCR-mediated low-density-lipoprotein cholesterol (LDL-C) and ALS. Fourth, two-sample MR was employed to analyze the relationship between LDL-C response to statins and ALS. The first and second MR tests explored the statins’ total effect and the third and fourth MR tests explored the peripheral LDL-C lowering effect of statins.

### 2.1. Exposure Data

As shown in Figure 1, four MR tests were conducted using different exposure sources. The first source was statin use, for which the GWAS summary statistics were obtained from Wu et al.’s study [27]. This study used self-reported regular medication data from the UK Biobank and classified the medications into 23 categories. These categories are based on the Anatomical Therapeutic Chemical (ATC) Classification System developed by the WHO, according to therapeutic, pharmacological and chemical properties [28]. A GWAS on statin use was performed with 73,475 cases and 216,910 controls. GWAS data on medication use were also utilized in recent MR studies, such as evaluations of the effect of opioid use on major depression [29,30,31]. The second exposure source was low-density-lipoprotein cholesterol (LDL-C), and summary statistics were obtained from a publicly available GWAS of 431167 European ancestry participants [32]. We accessed the GWAS summary statistics for LDL-C (GWAS ID: ebi-a-GCST90002412) through the IEU OpenGWAS database API http://gwas-api.mrcieu.ac.uk/ (accessed on 2 February 2023). The third source was HMGCR-mediated LDL-C, using SNPs within 100 kb windows from the gene HMGCR, which is associated with LDL cholesterol at a significance level of *p* < 5.0 × 10^−8^, as described by Huang et al. [26]. The fourth source was LDL-C response to statins, using the summary statistics from the two-stage GWAS performed by the Genomic Investigation of Statin Therapy Consortium (GIST) on 40914 statin-treated subjects of European ancestry [33].

### 2.2. Outcome Data: GWAS Summary Statistics for ALS

We used summary statistics from the publicly available GWAS summary statistics for ALS, including 20,806 cases and 59,804 controls of European ancestry (Figure 1 and Supplementary Table S1). The ALS cases were diagnosed as probable or definite ALS patients according to the EI Escorial criteria by a neurologist specializing in the field of ALS [34]. We accessed the GWAS summary statistics for ALS (GWAS ID: ebi-a-GCST005647) through the IEU OpenGWAS database API http://gwas-api.mrcieu.ac.uk/ (accessed on 2 February 2023).

### 2.3. MR Analysis

To assess the causal relationships between statin medication and ALS, we conducted two-sample MR and drug-target MR approaches (Figure 1). For the two-sample MR, independent SNPs identified in each GWAS that reached a certain threshold (*p*-value < 5 × 10^−8^ and r^2^ < 0.001) were selected as instrumental variants (IVs) for each exposure. In the MR analysis for assessing the relationship between statin response and ALS, we also chose a threshold of *p* < 5 × 10^−5^ in addition to *p* < 5 × 10^−8^ [33,35,36]. For the drug-target MR, we used 7 common SNPs (MAF > 1%) in low linkage disequilibrium (r^2^ < 0.30), associated with LDL cholesterol (*p*-value < 5.0 × 10^−8^) and located within ±100 kb windows from the HMGCR region, which has been shown in previous research [26]. SNPs absent in the outcome data were replaced by proxy SNPs (LD r^2^ values for proxies at 0.8 and a MAF threshold for aligning palindromes at 0.3) [37]. The strength of SNPs used as the instrument was assessed using the F-statistic, and we included SNPs with an F-statistic of >10 to avoid bias from weak instruments [38].

We used the inverse-variance-weighted (IVW) method as the principal MR analytical method to provide an overall estimate of the causal effect [39]. Sensitivity analysis, including weighted median [40] and MR–Egger regression methods [41], was also performed. Pleiotropy was evaluated via an MR–Egger intercept test [41]. To assess the robustness of the significant results, we also performed leave-one-out analyses to detect high influence points [42]. The results are expressed as odds ratios (ORs) with 95% confidence intervals (CIs). A *p*-value below 0.05 was defined as statistical significance. We performed all analyses using the ‘TwoSampleMR’ package version 0.5.6 in R software version 4.0.3 (R Foundation for Statistical Computing, Vienna, Austria. URL http://www.R-project.org/, accessed on 2 February 2023).

## 3. Results

### 3.1. Genetically Determined Statin Use Was Associated with a Higher Risk of ALS

**Figure 2:**
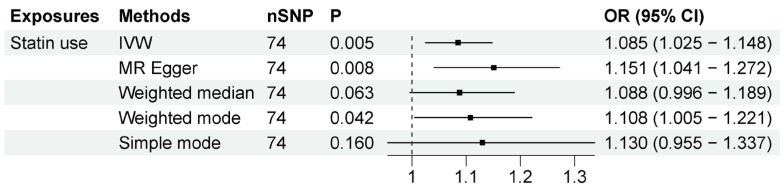
MR results between statin use and ALS risk. The number of SNP (nSNP) as instruments, odds ratio (OR) and 95% confidence interval (CI) are shown. Horizontal lines represent 95% CI and the diamond represents OR.

Statin use was associated with increased risk of ALS according to our two-sample MR analysis, which included 74 independent SNPs as instrumental variables (IVs). The results, shown in Figure 2, showed that statin use was associated with ALS (OR = 1.085, 95% CI = 1.025–1.148, *p* = 0.005) based on the inverse-variance-weighted method. The association was consistent with associations from the MR–Egger method (OR = 1.151, 95% CI = 1.041–1.272, *p* = 0.008) and other sensitivity analysis methods (Supplementary Data S1). The MR–Egger intercept analysis did not indicate horizontal pleiotropy (intercept, −0.005 ± 0.003, *p* = 0.166) (Supplementary Data S1). No high influence points were detected in the leave-one-out analysis (Supplementary Data S2). These data indicate that a genetic predisposition to statin use was associated with a higher risk of ALS.

### 3.2. Statin Use Potentially Mediates LDL-C Associated ALS Risk

**Figure 3:**
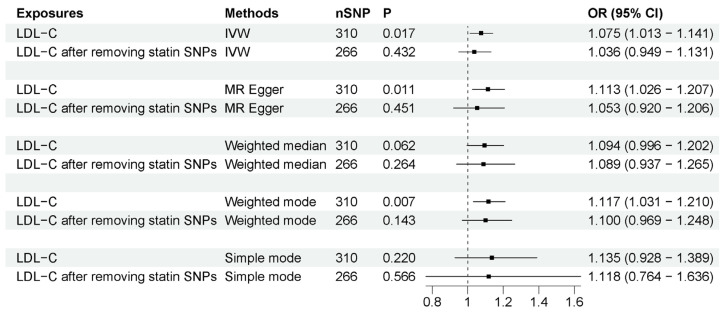
MR analysis of associations between LDL-C and ALS risk before and after removing SNPs associated with statin use. The number of SNP (nSNP) as instruments, odds ratio (OR) and 95% confidence interval (CI) are shown. Horizontal lines represent 95% CI and the diamond represents OR.

MR studies have identified that LDL-C is also associated with a higher risk of ALS [43,44]. To determine if statin use or LDL-C drives this association, we compared the associations before and after removing SNPs associated with statin use from LDL-C IVs. Before removing these IVs, as previous research indicated, LDL-C is indeed associated with an increased risk of ALS (OR = 1.075, 95% CI = 1.013–1.141, *p* = 0.017). However, after removing the IVs associated with statin use, the association between LDL-C and ALS was absent (OR = 1.036, 95% CI = 0.949–1.131, *p* = 0.432). The associations were generally consistent with different sensitivity analysis methods (Figure 3). The MR–Egger intercept analysis did not indicate horizontal pleiotropy (Supplementary Data S1). IVW leave-one-out analysis did not identify any single genetic variant with a high influence (Supplementary Data S2). These results suggest that the association between LDL-C and ALS risk may be potentially mediated by statin use.

### 3.3. Peripheral Effect of Statin Was Not Associated with ALS Risk

Since cholesterol cannot cross the BBB, the effects of statins need to be divided between two locations: the central nervous system and peripheral circulation. There are currently no SNPs that specifically represent statin function in the CNS. Meanwhile, the peripheral effects of statins can be evaluated using two methods: SNPs associated with a statin’s molecular target HMGCR-mediated LDL-C level (drug-target MR), and SNPs associated with the peripheral LDL-C response to statins (two-sample MR).

**Figure 4:**
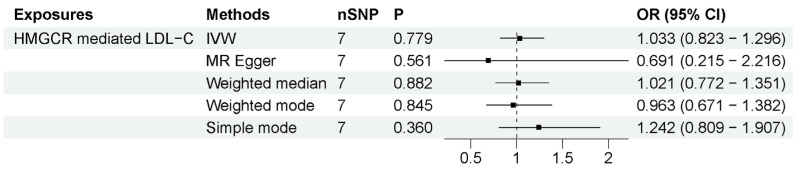
MR results between statin target HMGCR-mediated LDL-C and ALS risk. The number of SNP (nSNP) as instruments, odds ratio (OR) and 95% confidence interval (CI) are shown. Horizontal lines represent 95% CI and the diamond represents OR.

**Figure 5:**
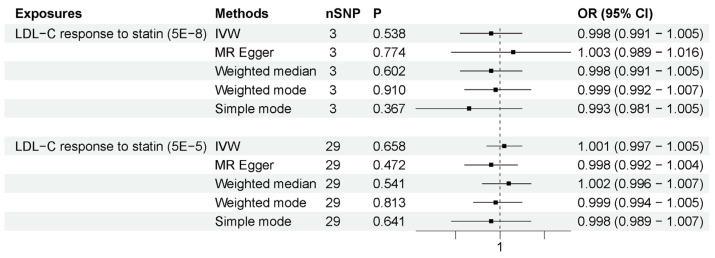
MR results for LDL-C response to statins and ALS risk. The number of SNP (nSNP) as instruments, odds ratio (OR) and 95% confidence interval (CI) are shown. Horizontal lines represent 95% CI and the diamond represents OR.

We employed these two methods to specifically explore the peripheral effects of statin use on ALS. (1) HMGCR-mediated peripheral LDL-C was not associated with ALS risk. The number of IVs to proxy HMGCR-mediated peripheral LDL-C was seven. As shown in Figure 4, the IVW method showed that HMGCR-mediated peripheral LDL-C had no association with ALS risk (OR = 1.033, 95% CI = 0.823–1.296, *p* = 0.779). The results showed that the association was consistent with different sensitivity analysis methods. The MR–Egger intercept analysis did not indicate horizontal pleiotropy (Supplementary Data S1). The leave-one-out analysis did not find a single genetic variant with a high influence (Supplementary Data S2). These data indicated that genetically determined HMGCR-mediated peripheral LDL-C was not associated with an increased risk of ALS. (2) Peripheral LDL-C response to statins was also not associated with an increased risk of ALS. The numbers of IVs to proxy LDL-C response to statin were 3 (*p*-value < 5 × 10^−8^) and 29 (*p*-value < 5 × 10^−5^). As shown in Figure 5, IVW results show that LDL-C response to statin was not associated with ALS risk (using IVs with *p*-value < 5 × 10^−8^: OR = 0.998, 95% CI = 0.991–1.005, *p* = 0.538; using IVs with *p*-value < 5 × 10^−5^: OR = 1.001, 95% CI = 0.997–1.005, *p* = 0.658). The association was consistent with different sensitivity analysis methods. The MR–Egger intercept analysis did not indicate horizontal pleiotropy (Supplementary Data S1). No single high-influence variant was identified in the leave-one-out analysis (Supplementary Data S2). Thus, circulation LDL-C response to statins was also not associated with ALS risk. In conclusion, the peripheral effects of statins showed no association with an increased risk of ALS, indicating that peripheral LDL-C lowering by statin use has little influence on the motor neurons in the CNS due to the BBB.

In summary, our study utilized an MR approach to uncover the potential causal effects of statins on increasing the risk of ALS. Our findings also shed light on the previously reported positive associations between LDL-C and ALS, which may be mediated by statin use. Furthermore, our research indicates that the increased risk of ALS associated with statin use is independent of peripheral cholesterol-lowering effects.

## 4. Discussion

Observational studies have indicated a potential association between statin use and ALS; however, these studies are constrained by confounding and reverse causality biases. In this study, we used a combination of MR methods to examine the causal relationship and the potential mechanisms of statins in ALS development. Our results provide genetic evidence that statin use may raise the risk of ALS, and that this risk is not due to reduced peripheral circulation of LDL-C. Our findings extend previous observational [14,15,16,17] and animal studies [45,46] suggesting that statins may increase ALS risk and indicating that the mechanism may occur in the CNS. Given that statins are widely prescribed, these results not only facilitate our understanding about the mechanism and etiology of ALS, but also have important implications for clinical decision-making and management for ALS patients.

Our study has clarified to some extent the relationship between statins, cholesterol metabolism and ALS. Due to the BBB, which tightly regulates the movement of cholesterol between blood and the brain, CNS is independent from peripheral tissues in terms of lipid metabolism. The BBB also affects drug delivery to the CNS [47]. The liposolubility of statins may increase their ability to cross the BBB [48]. Therefore, analyzing the pharmacological effects of drugs on the CNS is complex and difficult, and it is necessary to separate the direct effects on the CNS from the secondary effects of peripheral circulation. This study used different GWAS and MR methods to identify the potential effects of statins and mechanisms. The results provide evidence that statin use is associated with an increased risk of ALS and contributes to a higher risk of ALS related to LDL-C. The peripheral lipid-lowering effects of statins in circulation were not found to be causally related to the risk of ALS, indicating that the direct effects of statins in CNS mediate the higher risk of ALS. The study also helps to identify the role of blood LDL-C in ALS. Previous studies have shown that high levels of circulating LDL-C increase the risk of ALS using MR methods [43,44], while paradoxically, cholesterol levels are elevated during the course of the disease and higher serum cholesterol is associated with longer survival [23,49,50,51]. The results of this study suggest that the causality between LDL-C and ALS is not attributed to blood LDL-C levels themselves but may be mediated by clinical statin prescription. This finding resolves the above contradiction and suggests that peripheral cholesterol dyshomeostasis in ALS may be a consequence or epiphenomenon.

The cell biology of ALS involves death of motor neurons, while cancer is characterized by uncontrolled cell proliferation [11,12,13]. Even though the pathogenic mechanisms of statin in the pathophysiology of ALS remain unclear, lessons can be learned from cancer research. Statins inhibit the process that converts HMG-CoA to mevalonate, resulting in the synthesis of cholesterol and isoprenoids. Thus, statins not only decrease cholesterol levels, but also inhibit mevalonate-mediated cholesterol-independent effects (such as isoprenoid level reduction). We deduced that statins can increase ALS risk by both cholesterol-mediated and non-cholesterol-mediated mechanisms. (i) Cholesterol-mediated mechanisms: Cholesterol-enriched microdomains known as lipid rafts are essential for activating pro-survival signals. Caveolin, a membrane protein, needs to localize to lipid rafts and organizes many signal transduction proteins to activate intracellular survival signaling pathways (such as PI3K-AKT and JAK–STAT pathways) [11,52,53]. Statins are thought to interfere with the organization of lipid rafts and inhibit these pro-survival pathways due to their effects on cholesterol homeostasis [11,54]. This supposed molecular mechanism is supported by the fact that caveolin can promote neurotrophic signaling, leading to enhanced neuronal survival and inhibiting ALS progression, and is being developed as a therapy for ALS (US patent no. 8969077B2) [55,56,57]. (ii) Non-cholesterol-mediated mechanisms: The statin-suppressed mevalonate pathway can also support cell survival via non-cholesterol-mediated mechanisms. Mevalonate itself can facilitate cell survival, such as via cyclin-dependent kinase 2 (CDK2) activation [11]. Isoprenoids downstream mevalonate can facilitate pro-survival pathways, such as Ras and Rho signaling [58,59] and YAP/TAZ signaling [60,61,62,63]. Thus, statins might suppress motor neuron survival by reducing mevalonate and pro-survival signals mediated by downstream isoprenoids. In addition, statins also seem to induce apoptosis by activating pro-apoptosis pathways, such as FOXO3a and Bcl-2 pro-apoptotic pathways [64,65]. Understanding these mechanisms is instrumental in statin use for ALS patient management and therapeutics, and the potential mechanisms mentioned above need to be validated in the future.

Our findings have important clinical implications for improving clinical practice and precision medicine. This study identified statin medication may represent a new type of risky exposure (medical exposure) for ALS development. Among all exposures, including environmental and lifestyle factors, medication exposure is the most closely related to clinical practice. If statins increase the risk of ALS, caution should be taken in using statins in cases of cholesterol dysregulation and ALS. If statin use is deemed necessary, hydrophilic statins with limited ability to cross the BBB, such as pravastatin, may be a viable option. In the future, we need to explore the distinct effects of hydrophilic and liposoluble statins on ALS risk through various animal experiments and population observations; In addition, identifying other risky medication exposures for ALS is also critical to achieve precision medicine in ALS patient management.

Our study also has some limitations. Firstly, it is extremely difficult to completely rule out horizontal pleiotropy and alternative direct causal pathways, which is a limitation faced by all MR studies. We emphasize that our present results should be interpreted with caution. However, we conducted the MR–Egger intercept test which did not show horizontal pleiotropy. Secondly, the database may have biases. SNP associations were identified only in European populations aged 38–73 years, mostly from UKB, which may have referral bias or survival bias and may not be generalizable to other populations [66]. The dose and duration of drug exposure may vary among individuals and may change dynamically, making it difficult to accurately measure medication exposure [67]. Validation using non-UKB original data is still needed, but such data are not yet available. Thirdly, the relationship between statin and LDL-C is complex. High LDL-C levels can influence the decision to take statins, and statins can lower LDL-C levels, which can impact patient behavior. Additionally, statins can regulate LDL-C in both the CNS and periphery. Thus, there is no perfect approach to completely eliminate confounding from these tight interactions, and even RCTs cannot rule out this type of confounding [68]. Despite using genetically predicted data to reduce confounding and promote caution, and despite the success of detecting causal associations between medications and diseases via a GWAS of medication use [29,30,31], we still consider it to be more rigorous to combine different levels of reported evidence or in the future and consider spatial–temporal variation. If strong proxies for CNS-specific statin function biomarkers become available in the future, similar to the circulating biomarker LDL-C level, we may be able to directly assess the role of statins in the CNS, including in motor neurons in situ [25].

## 5. Conclusions

In conclusion, using different MR approaches, our study showed that genetically predicted statin use potentially increased the risk of ALS, which was not attributed to LDL-C lowering effect in the peripheral circulation. That implies that among the modifiable risky exposures, statin medication may represent a new type of risky exposure (medical exposure) for ALS development. These findings may suggest the underlying biological mechanisms of ALS occurrence and inform prevention and intervention strategies in ALS management. In the future, we hope further experimental research and more strictly designed and different population-based GWASs can be used to validate our results, and clarifying the distinct effects of hydrophilic and liposoluble statins on the risk of developing ALS is key to promote precision medicine.

## Data Availability

Exposure and outcome GWASs are available in previous research, which has been shown in the section of methods.

## Supplementary Materials

The following supporting information can be downloaded at: https://www.mdpi.com/article/10.3390/biomedicines11051359/s1, Data S1. MR results; Data S2. Leave one out sensitivity analysis.
