# Supplementary material for: Mendelian Randomization Analysis Reveals Statins Potentially Increase Amyotrophic Lateral Sclerosis Risk Independent of Peripheral Cholesterol-Lowering Effects
Source: Biomedicines. 2023 May 4;11(5):1359. doi: 10.3390/biomedicines11051359 (PMC10216016; doi:10.3390/biomedicines11051359)

1. Leave-one out sensitivity analysis between statins and ALS

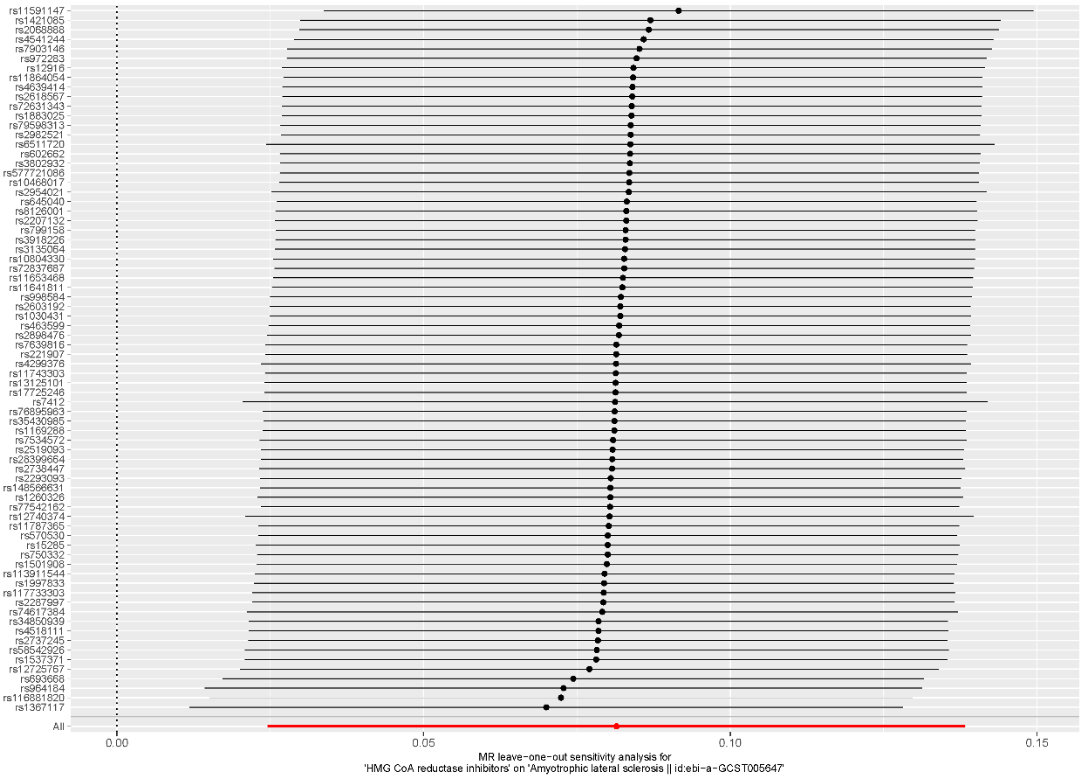

2. Leave-one out sensitivity analysis between LDL-C and ALS

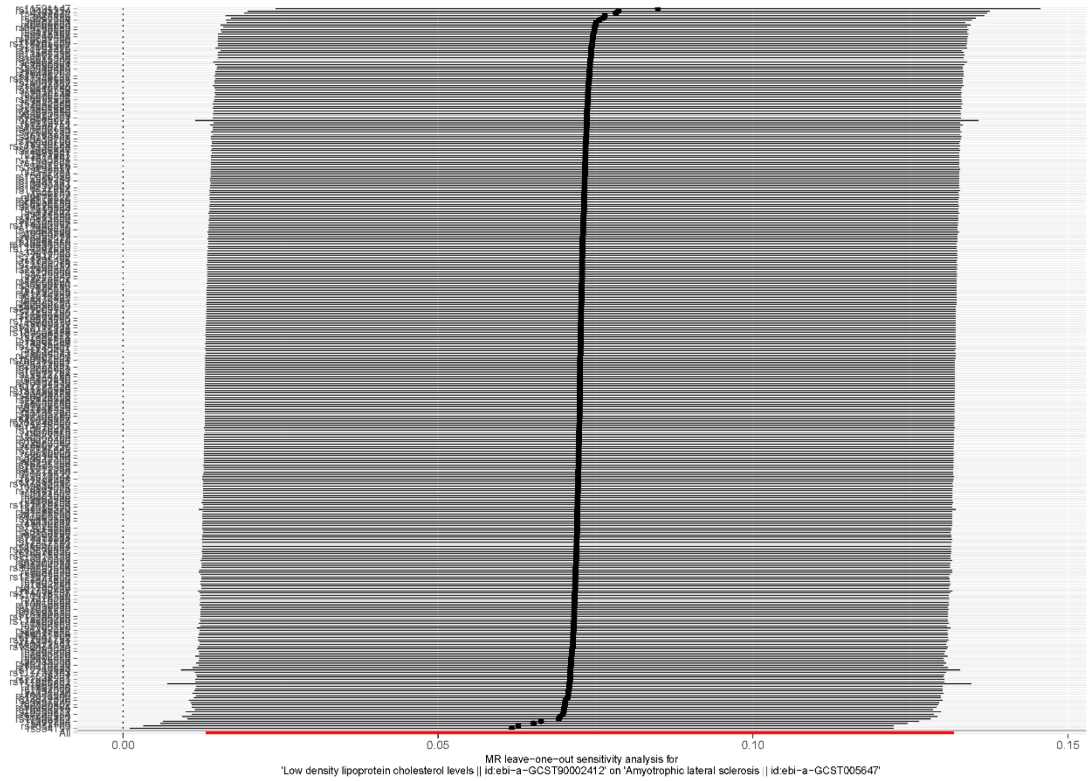

3. Leave-one out sensitivity analysis between LDL-C (after removing IVs associated with statin use) and ALS

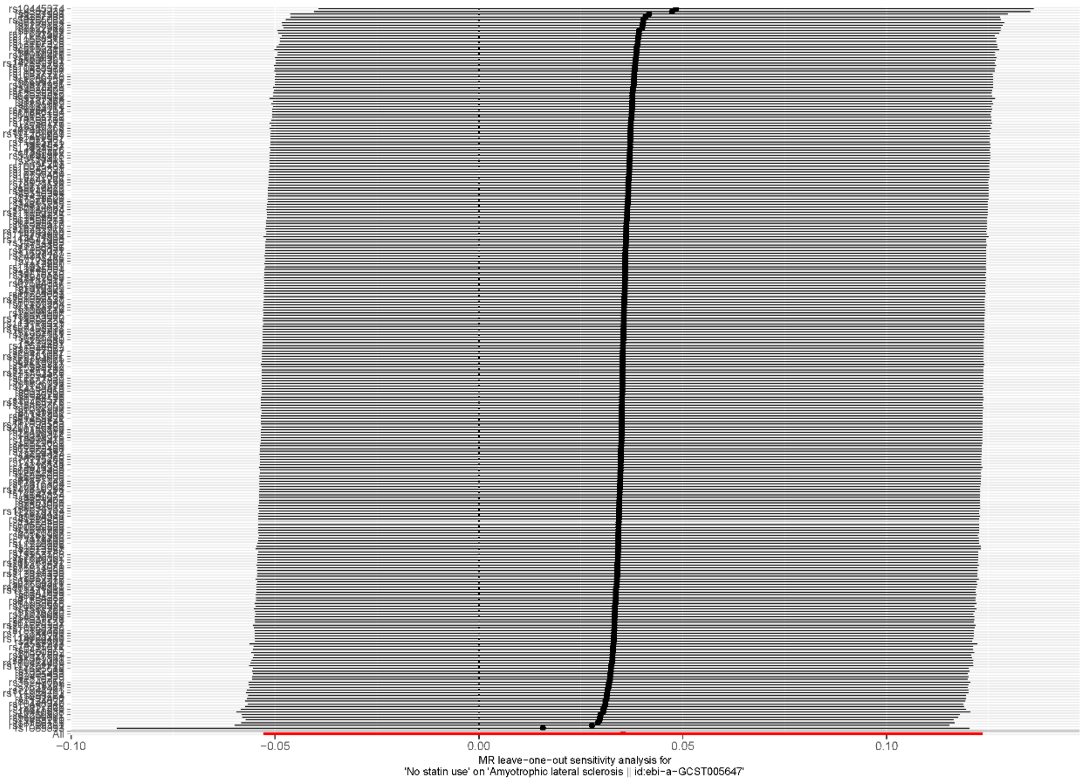

4. Leave-one out sensitivity analysis between HMGCR mediated LDL-C and ALS

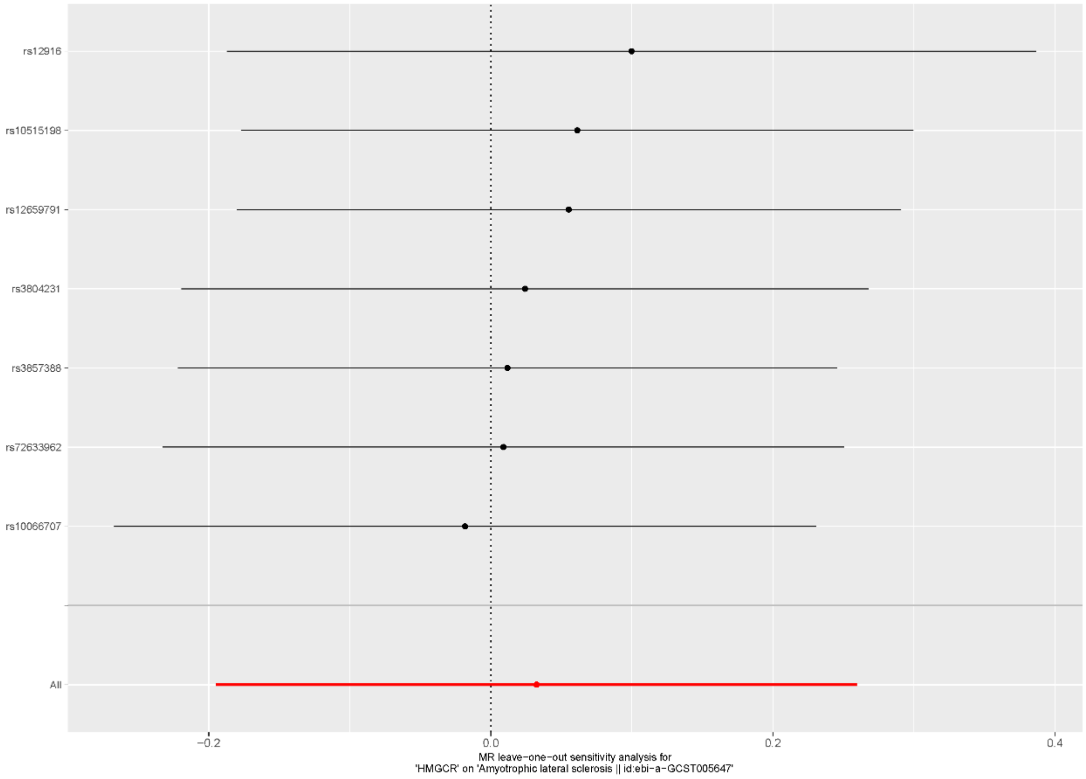

5. Leave-one out sensitivity analysis between LDL-C response to statins (P < 5E-8) mediated LDL-C and ALS

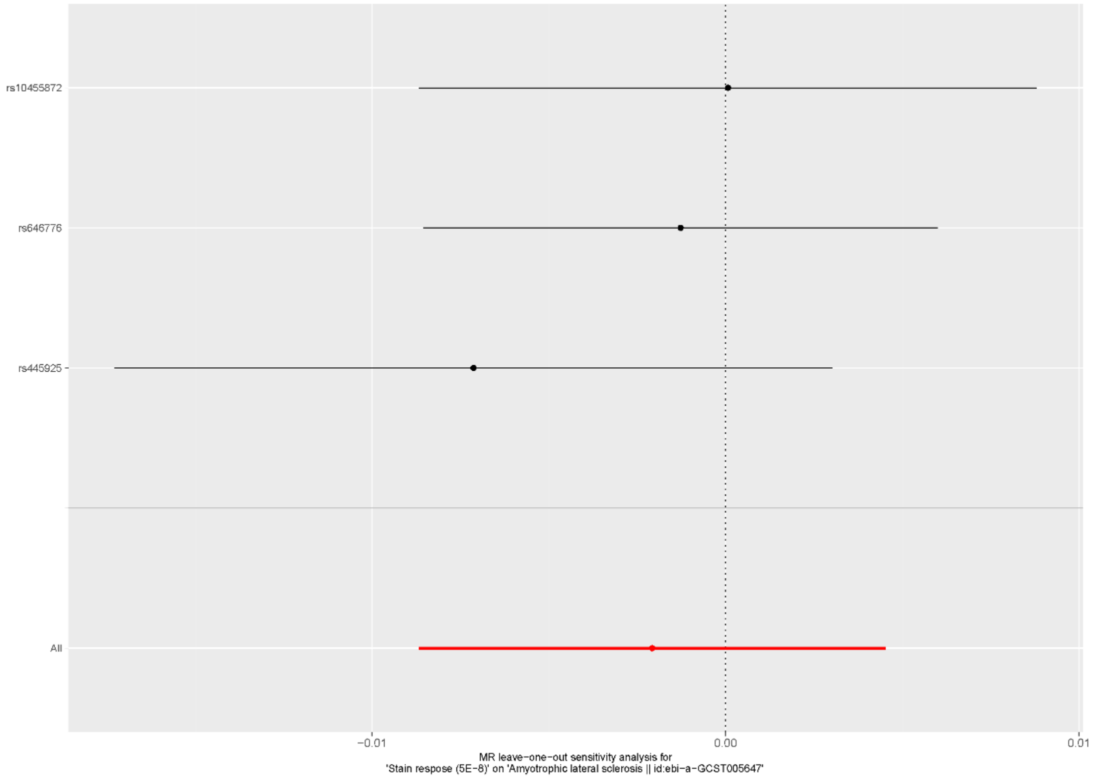

6. Leave-one out sensitivity analysis between LDL-C response to statins (P < 5E-5) mediated LDL-C and ALS

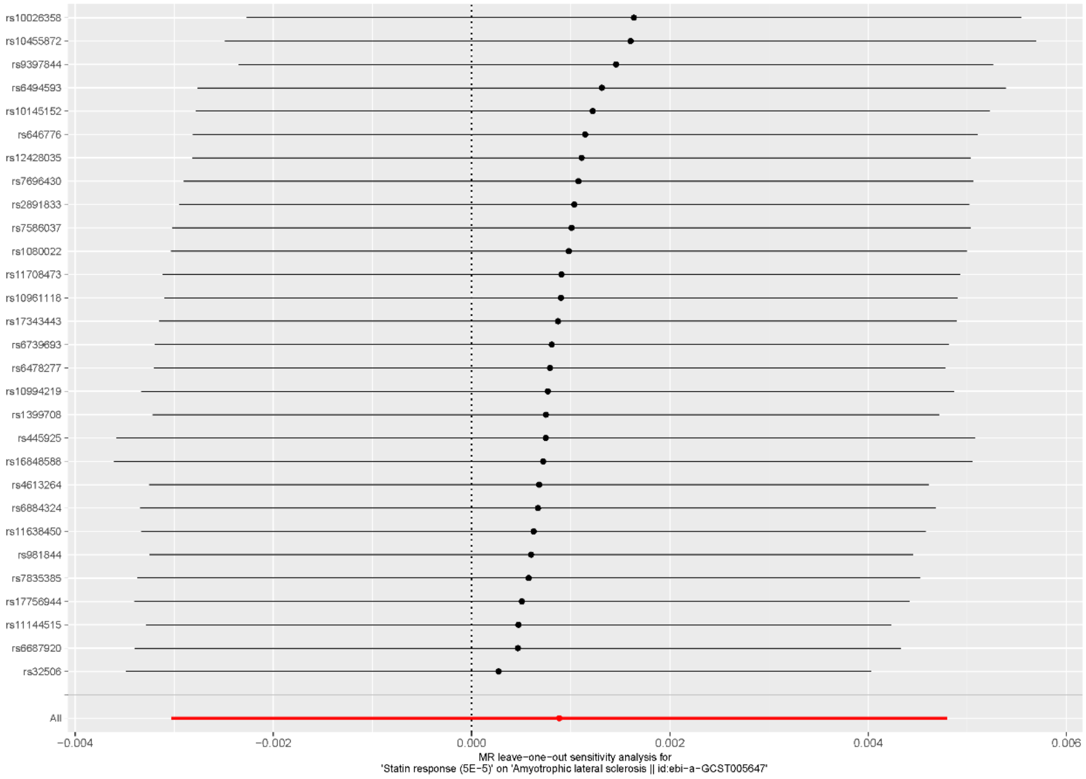

Supplement: Supplementary file 1 [file biomedicines-11-01359-s001.zip › Supplementary Data 2. Leave one out sensitivity analysis.pdf]
